# Meningioma with ring enhancement on MRI: a rare case report

**DOI:** 10.1186/s12880-021-00555-x

**Published:** 2021-02-10

**Authors:** Miao Wang, Zhongke Wang, Peng Ren, Xiaoqing Zhang, Shiyong Liu

**Affiliations:** Department of Neurosurgery, Xinqiao Hospital, Army Medical University, 400037 Chongqing, China

**Keywords:** Meningioma, Magnetic resonance imaging, Ring enhancement

## Abstract

**Background:**

Meningiomas typically manifest on magnetic resonance imaging (MRI) as iso- to hypointense on T1-weighted imaging and iso- to hyperintense on T2-weighted imaging. After contrast administration, they usually homogeneously enhance and exhibit a visible dural tail. Meningiomas with atypical findings may be misdiagnosed.

**Case presentation:**

We report a 50-year-old female patient with a pathologically diagnosed fibrous meningioma (World Health Organization grade I) that exhibited ring enhancement on MRI.

**Conclusions:**

Meningiomas may rarely present with ring enhancement on MRI. The natural history and mechanisms of cystic degeneration and enhancement in the various types of meningioma require further study.

## Background

Meningiomas are a common primary intracranial tumour andaccount for 13.0–19.0 % of all surgically treated intracranial tumours [1, 2]. Most cases are benign. The preoperative diagnosis of meningioma mainly depends on computed tomography (CT) or magnetic resonance imaging (MRI). On MRI, meningiomas typically appear iso- to hypointense on T1-weighted imaging (T1WI) and iso- to hyperintense on T2-weighted imaging (T2WI). After contrast administration, they usually homogeneously enhance and exhibit a visible dural tail [3]. However,histologically confirmed meningiomas may show atypical MRI findings [4] including inhomogeneous enhancement and cystic degeneration; ring enhancement is especially rare. We report a pathologically confirmed meningioma that exhibited ring enhancement after contrast administration.

## Case presentation

**Fig. 1:**
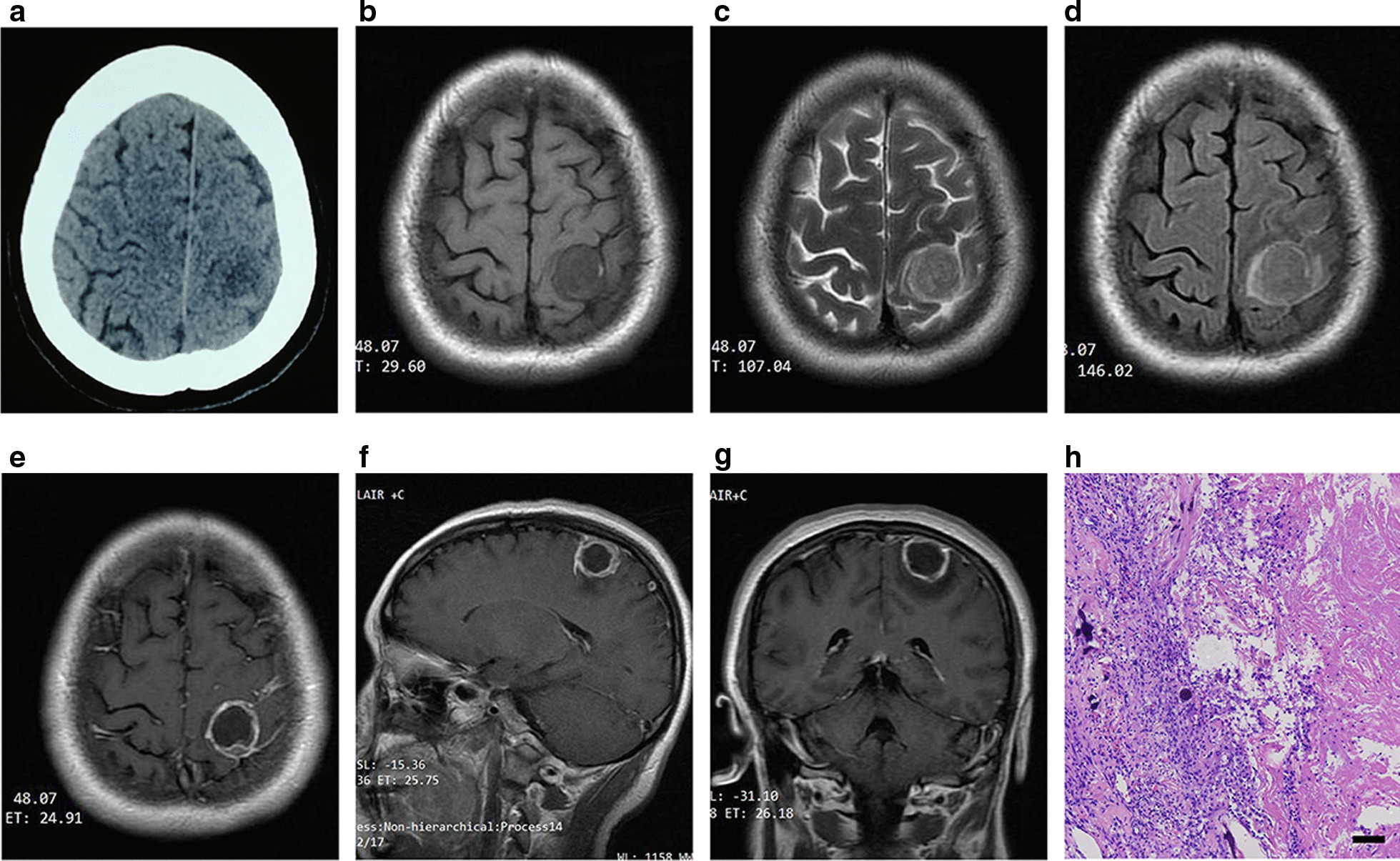
Head CT showed an abnormal left frontal-parietal hypodense lesion (**a**). T1 (**b**), T2 (**c**) and T2-FLAIR (**d**) showing lesions that appeared iso- to hypointense on T1WI and iso- to hyperintense on T2WI. Signal hyperintensity was present in the surrounding brain on T2WI and fluid attenuated inversion recovery sequences, consistent with mild oedema. On axial enhanced T1 (**e**), sagittal (**f**) and coronal (**g**) ring enhancement was seen with a slightly thickened cystic wall and dural tail sign. Microscopic examination (h) confirmed fibrous meningioma with extensive central degeneration and necrosis of central tumour cells (haematoxylin/eosin [H&E]). Scale bar: 100 µm

A 50-year-old woman with no significant medical or family history presented with a headache of 15 days duration. Physical and neurological examinations were unremarkable. Routine blood testing was normal. Head CT showed an abnormal left frontal-parietal hypodense lesion that appeared iso- to hypointense on T1WI and iso- to hyperintense on T2WI. Signal hyperintensity was present in the surrounding brain on T2WI and fluid attenuated inversion recovery sequences, consistent with mild oedema. On contrast-enhanced T1WI, ring enhancement was seen with a slightly thickened cystic wall and dural tail sign. Cerebrospinal fluid was visible between the lesion and the underlying graymatter, suggesting an extra-axial lesion (Fig. 1). After hospital admission, no other systemic tumours were found on screening examinations.

The patient underwent a left frontal-parietal craniotomy for tumour resection. Intraoperatively, the tumour arose from the left parietal dura mater; no skull involvement was found. The tumour and adjacent dura mater within 1 cm of the tumour margin were removed; necrotic tissue was visible within the tumour. Histopathological examination confirmed fibrous meningioma with extensive central degeneration and necrosis (World Health Organization grade I). The patient recovered well without neurological deficits.

## Discussion and conclusions

Meningiomas are the most common non-glial cell-derived brain tumour. Their annual incidence is approximately 6 per 100,000 persons, and the male-to-female ratio is approximately 1:2 [5]. Most meningiomas have typical CT and MRI manifestations and are relatively easy to diagnose based on imaging data. However, some present with atypical findings, such as inhomogeneous enhancement and cystic degeneration on contrast-enhanced T1WI. Meningiomas with ring enhancement have rarely been reported [6].

Even if contrast-enhanced brain CT provide useful information of intracranial disorders, enhanced brain MRI represents the gold standard technique for the assessment and characterization of malignant and benign lesions [7]. Common ring-enhancing intracranial lesions include glioblastoma, brain abscess and metastatic tumour. The ring enhancement in glioblastoma manifests as an irregular or incomplete cystic wall with uneven wall thickness, and the cystic wall is tension-free; nodular or irregular enhancement is often found within or around the ring; the use of Gradient Echo or Susceptibility weighted imaging is helpful in the differential diagnosis due to their sensitivity in detecting blood products: the presence of an intralesional hemorrhage, in fact, can support the diagnosis of a GBM. Brain abscess ring enhancement is characterized by a thin and uniform cystic wall with relatively smooth inner and outer surfaces and the presence of tension. The ring enhancement of metastatic tumours is related to the degree of central tumour necrosis: tumours with less central necrosis have relatively thick-walled ring enhancement with uneven thickness, while patients with more central necrosis show thin-walled ring enhancement with tension. Diffusion-weighted imaging (DWI) can be used to differentially diagnose brain abscess from tumour necrosis. In DWI, the signal intensity of brain abscess is higher than that of brain parenchyma, presenting high signal, while tumour necrosis shows low signal. It is difficult to make an imaging diagnosis of meningioma if the lesion exhibits ring enhancement. In our patient, preoperative MRI showed ring enhancement and a cystic wall with uniform thickness; the inner and outer surfaces of the wall were not smooth, suggesting tumour-like ring enhancement. On T2WI, cerebrospinal fluid was visualized between the lesion and the surrounding brain, and there was mild oedema in the peritumoural brain tissue. In addition, a dural tail was visible on sagittal and coronal contrast-enhanced images. The combination of these imaging features led to a preoperative diagnosis of meningioma.

As reported in previous studies, ring enhancement in meningiomas may be related to necrosis, haemorrhage and cystic degeneration in the centre of the tumour [8, 9]. Intratumoural cystic degeneration of solid tumours may be caused by degenerative changes, tumour ischaemia, intratumoural haemorrhage [10, 11], and tumour secretion, as Michaud and Cagne reported that the transsudation of low-protein fluid in some well vascularized meningiomas, could be responsible for the cyst formation [12]. Moreover, necrosis is more likely to occur during rapid tumour growth. In this case, we found a slow growing meningioma; thus, the cause of necrosis and ring enhancement is unclear. Our patient was finally diagnosed with fibrous meningioma based on pathological findings. Fibrous meningiomas are more prone to cystic degeneration than other types [13]. The natural history and mechanisms of cystic degeneration and enhancement in the various types of meningioma require further study.

## Data Availability

Not applicable.

## Abbreviations

CT: Computed tomography
MRI: Magnetic resonance imaging
T1WI: T1-weighted imaging
T2WI: T2-weighted imaging
